# Rat visceral yolk sac cells: viability and expression of cell markers during maternal diabetes

**DOI:** 10.1590/1414-431X20154739

**Published:** 2015-07-10

**Authors:** M.B. Aires, J.R.A. Santos, K.S. Souza, P.S. Farias, A.C.V. Santos, E.T. Fioretto, D.A. Maria

**Affiliations:** 1 Departamento de Morfologia, Universidade Federal de Sergipe, São Cristóvão, SE, Brasil; 2 Departamento de Enfermagem, Universidade Federal de Sergipe, São Cristóvão, SE, Brasil; 3 Laboratório de Bioquímica e Biofísica, Instituto Butantan, São Paulo, SP, Brasil

**Keywords:** Visceral yolk sac, Diabetes, Embryo, Pregnancy, Cell markers

## Abstract

The function of the visceral yolk sac (VYS) is critical for embryo organogenesis until final fetal development in rats, and can be affected by conditions such as diabetes. In view of the importance of diabetes during pregnancy for maternal and neonatal health, the objective of this study was to assess fetal weight, VYS cell markers, and viability in female Wistar rats (200-250 g) with induced diabetes (alloxan, 37 mg/kg) on the 8th gestational day (gd 8). At gd 15, rats from control (n=5) and diabetic (n=5) groups were anesthetized and laparotomized to remove the uterine horns for weighing of fetuses and collecting the VYS. Flow cytometry was used for characterizing VYS cells, and for determining mitochondrial activity, cell proliferation, DNA ploidy, cell cycle phases, and caspase-3 activity. Fetal weight was reduced in the diabetic group. Expression of the cell markers CD34, VEGFR1, CD115, CD117, CD14, CCR2, CD90, CD44, STRO-1, OCT3/4, and Nanog was detected in VYS cells in both groups. In the diabetic group, significantly decreased expression of CD34 (P<0.05), CCR2 (P<0.001), and OCT3/4 (P<0.01), and significantly increased expression of CD90 (P<0.05), CD117 (P<0.01), and CD14 (P<0.05) were observed. VYS cells with inactive mitochondria, activated caspase-3, and low proliferation were present in the rats with diabetes. Severe hyperglycemia caused by maternal diabetes had negative effects on pregnancy, VYS cell viability, and the expression of cell markers.

## Introduction

Maternal diabetes is a factor predisposing to embryonic lethality and congenital abnormalities (1). Reports have documented placental defects in the junctional and labyrinth zones in diabetic rats (2,3). In addition to the chorioallantoic placenta, rodents also have a well-developed visceral yolk sac (VYS) that acts as an active region for metabolic exchange and nutrition uptake (4,5).

The VYS consists of an endodermal epithelium, a basement membrane, and a mesoderm with capillaries (4). In rodents, the VYS does not degenerate once the placenta becomes fully functional but continues to contribute at some level to the absorption and digestion of nutrients until just before parturition (4,6). In fact, evidence based on mitochondrial metabolic function indicates that the VYS becomes a supporting tissue that remains active during the days following the establishment of placentation from gestational day 12 (gd 12) onwards (7). However, there is no information about the effects of severe hyperglycemia as induced by alloxan on VYS cells at gd 15 in rats.

The yolk sac is considered the first site of hematopoiesis during mammalian development (8). Moreover, it is a source of mesenchymal stem cells (MSCs), which can differentiate into osteoblasts, chondrocytes, and adipocytes in mice (9), and into adipocytes and neurons in humans (10,11). Thus, the VYS seems to be critical during organogenesis until final fetal development in rats, and could also be an important source of MSCs. In view of the importance of diabetes during pregnancy for maternal and neonatal health, the objective of this study was to assess fetal weight, VYS cell viability, and cell marker expression levels in diabetic female rats at gd 15.

## Material and Methods

### Animals and diabetes induction

Twenty-five female and five male adult Wistar rats weighing 200-250 g were used for mating (Ethics Committee approval from Universidade Federal de Sergipe, CEPA #86/2011). The morning when spermatozoa were found in the vaginal smear was designated gestational day 1 (gd 1). Diabetes was induced by a single injection of alloxan monohydrate (37 mg/kg, *iv*) in saline solution on gd 8, after 12 h of starvation. Animals in the control group received an identical volume of sterile saline solution. Animals with a blood glucose level >200 mg/dL at gd 10 (Accu-Chek Performa test strips, Roche Diagnostics, Brazil) were included in the diabetic group. On gd 15, rats from the control (n=5) and diabetic (n=5) groups were anesthetized, exsanguinated, and laparotomized to remove the uterine horns for collecting the placentas and weighing fetuses.

### Yolk sac sampling

Placentas were transferred to a Petri dish on ice filled with sterile saline solution. The VYS was dissected with the aid of a lighted head-mounted magnifying glass and dissociated mechanically, before being filtered through 30-µm sterile filters, and added to a freezing solution (10% DMSO, 60% RPMI-1640, 30% fetal bovine serum); samples were stored in a -70°C freezer.

The cells obtained were washed with phosphate-buffered saline (PBS), pooled, and centrifuged at 274 *g* for 10 min. The supernatant was discarded, and the pellet was resuspended in 5 mL PBS at 10^6^ cells/mL. Duplicate samples were subjected to flow cytometry (BD FACScalibur; Becton Dickinson, USA) to characterize VYS cells, and to determine mitochondrial activity, DNA ploidy, cell cycle phases, cell proliferation, and caspase-3 activity.

### Characterization of VYS cells

The VYS cells were rinsed with PBS and resuspended in 300 μL 0.03 g/L trypsin, 10 mM Tris, pH 8.0. Then, the cells were stained with antibodies to CD34 (Santa Cruz Biotechnology, USA), VEGFR-1 (Sigma-Aldrich, USA), CD115, CD117 (Abcam, USA), CD14, CCR2 (Santa Cruz Biotechnology), and CD90, CD44, STRO-1, OCT3/4, and Nanog, all from Abcam, at a concentration of 1 μg/mL at 4°C for 30 min. The corresponding isotype control antibody was used as a negative control, and goat anti-mouse IgG (H/L):FITC was used as a secondary antibody (AbD Serotec, USA). The cells were pelleted, washed twice with PBS and fixed with 1% paraformaldehyde. Fluorescence-activated cell sorting (FACS) analysis was performed on a BD FACSCalibur flow cytometer using CellQuest software (Becton Dickinson), and Win MDI 2.9 software (http://winmdi.software.informer.com/) was used for the acquisition of data and analysis of histograms. A minimum of 10,000 events was counted for each analysis.

### Measurement of mitochondrial transmembrane potential (Δψm)

Rhodamine 123 (Rho123) is a lipophilic fluorescent dye that has been used to estimate the electrical potential across the inner mitochondrial membrane (Δψm). It accumulates in the inner mitochondrial membrane and mitochondrial matrix because of its charge and solubility and is able to produce images of high fluorescence for live mitochondria (12). Here, the Δψm was measured using a Rho 123 assay monitored by flow cytometry. VYS cells (10^6^ cells/mL) were incubated in a Rho 123 solution (100 mg/mL) diluted in DMSO (5 μg/mL) in a 5% CO_2_ incubator for 30 min. After being washed with PBS, the cells were analyzed using a FACScan flow cytometry system (Becton Dickinson). A minimum of 10,000 events was counted for each analysis.

### Proliferation index by 5,6-carboxyfluorescein diacetate succinimidyl ester (CFSE-DA)

Determination of cell proliferation using the CFSE-DA labeling method was adapted from a previously described protocol that allows for the direct detection of single proliferating cells, and facilitates the quantification of cell division by flow cytometry, according to respective CFSE-DA-dilutions (13). The principle of cell proliferation analysis is as follows: CFSE-DA is divided equally between daughter cells following cell division, and the intensity of fluorescence is half that of the parental generation. Thus, in a cell population undergoing proliferation, fluorescence intensity declines by 50% in the next generation. CFSE-DA flow cytometric data files were analyzed using CellQuest acquisition/analysis software (Becton Dickinson). Fifty thousand events were collected, and the proliferation index was determined using ModFitLT 2.0 software (Proliferation Wizard Methods; Becton Dickinson).

### Cell cycle analysis

VYS cells were rinsed with PBS and resuspended in 300 μL 0.03 g/L trypsin, 10 mM Tris, pH 8.0. After 15-min incubation at room temperature, 100 μL of neutralization solution (0.5 g/L trypsin inhibitor, 0.1 g/L RNase A and 1.2 g/L spermidine) was added and incubation continued for 15 min. After centrifugation, cells were resuspended in 300 μL PBS and fixed by the addition of 70% ice-cold ethanol. Prior to analysis, cells were incubated with 1.8 μg/mL propidium iodide solution (Sigma-Aldrich) and incubated in the dark for 30 min. Flow cytometric analysis was performed using a FACSCalibur flow cytometer (Becton Dickinson). The DNA contents in the cell cycle phases (sub-haploid, G0/G1, S and G2/M) were analyzed using CellQuest and ModFitLT 3.2 software (Becton Dickinson).

### Caspase 3 activity

The pellet of VYS cells was resuspended in PBS at a concentration of 5×10^5^ cells/mL and incubated for 1 h at 4°C with 1 μL of specific anti-active caspase-3 antibody with phycoerythrin (PE; Abcam), in the absence or presence of an Ac-Asp-Glu-Val-Asp-OH-specific inhibitor (Abcam). After this, cells were centrifuged at 428 *g* for 10 min, rinsed twice with cold PBS and fixed with 1% paraformaldehyde. Flow cytometer settings were established using unstained cells. Cells were gated by forward-scattering to eliminate debris. To eliminate the possible autofluorescence of VYS, the contribution of unstained cells was removed from the measurement channel. A minimum of 10,000 events was counted for each analysis. Cells were evaluated for cell surface protein expression using a FACSCalibur flow cytometer (Becton Dickinson) using CellQuest and Win MDI 2.9 software (Becton Dickinson) to create the histograms.

### Statistical analysis

Data are reported as means±SE. Comparisons between control and diabetic groups for mean fetal weight and maternal glycemia were performed using Student’s *t*-test and by one-way analysis of variance followed by Tukey’s multiple comparison test for significant differences between groups for the characterization of VYS cells, measurement of Δψm, proliferation index, DNA ploidy, cell cycle analysis, and caspase-3 activity. A value of P<0.05 was considered to be significant.

## Results

### Maternal glycemia and fetal weight

In control rats, normoglycemia based on blood glucose level was verified at gd 10 (111.0±4.4 mg/dL), which was significantly different from the diabetic group (452.0±31.5 mg/dL; P<0.0001). At gd 15, hyperglycemia was confirmed in the diabetic group (430.0±87.6 mg/dL; P<0.001) compared with normoglycemia in the control group (95.4±2.1 mg/dL). Fetal weight was reduced significantly in the diabetic group (123.9±5.2 mg) compared with the control group (137.2±3.5 mg, P<0.05).

### Expression of cell markers by VYS cells

**Figure 1:**
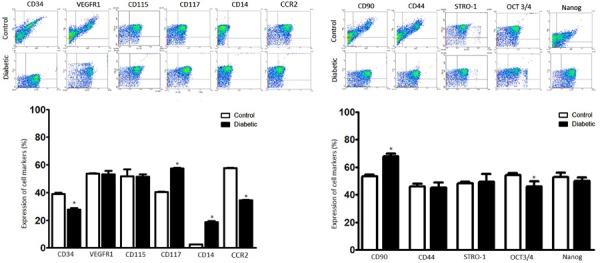
Representative flow cytometry plots of VYS cells from the control and diabetic groups. These were stained with markers for hematopoiesis (CD34, VEGFR1, CD117), monocytes/macrophages (CD115, CD14, CCR2), stromal tissue (CD90, CD44), and pluripotency (STRO-1, OCT3/4, Nanog). The expression of CD34, CCR2, and OCT3/4 was significantly reduced in the diabetic group. The histograms show the increased expression of CD90, CD117 and CD14 in the diabetic group compared with the control. Results are reported as the means±SE of cells collected from five yolk sacs per animal of the control (n=5) and diabetic groups (n=5) analyzed in duplicate. *P<0.05 (ANOVA and Tukey's multiple comparison test).

Expression of the VYS cell markers CD34, VEGFR1, CD115, CD117, CD14, CCR2, CD90, CD44, STRO-1, OCT3/4, and Nanog were detected on VYS cells in both the control and diabetic groups (Figure 1). The expression levels were significantly reduced in the diabetic group: CD34 (39.2±0.8 *vs* 27.8±1.0, P<0.05), CCR2 (57.7±0.2 *vs* 34.5±0.5, P<0.001), and OCT3/4 (54.2±0.8 *vs* 47.9±0.6, P<0.01). Significantly increased expression levels of CD90 (53.4±0.9 *vs* 67.9±1.5, P<0.05), CD117 (40.5±0.3 *vs* 57.6±0.3, P<0.01) and CD14 (2.5±0.1 *vs* 18.7±0.7, P<0.05) were measured in the diabetic compared with the control groups, respectively.

### Mitochondrial transmembrane potential and caspase 3 activity

**Figure 2:**
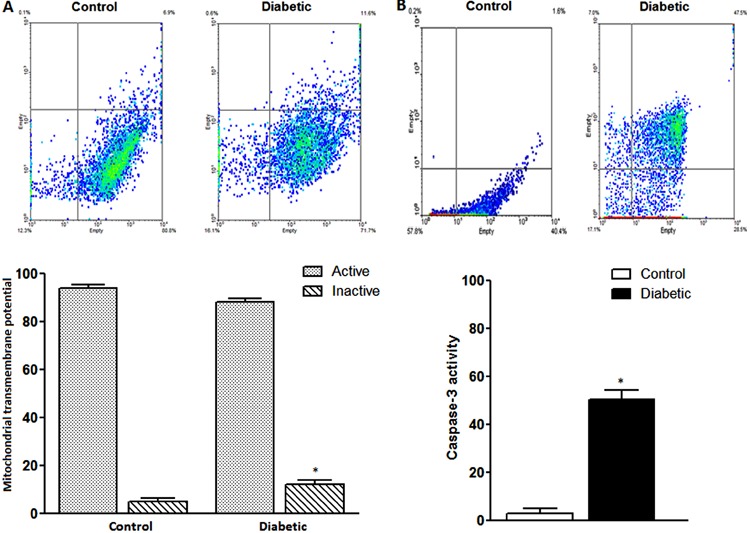
*A*, Flow cytometry plots of visceral yolk sac (VYS) cells from control and diabetic groups stained with rhodamine 123. The histograms show an increased number of VYS cells with inactive mitochondria (unviable cells) in the diabetic group and a similar number of cells with active mitochondria (viable cells) in both groups. *B*, Representative flow cytometry plot of activated caspase-3 in VYS cells of control and diabetic groups. The histogram shows a large number of activated caspase-3-expressing VYS cells in the diabetic group. Results are reported as the means±SE of VYS cells collected from five yolk sacs per animal of the control (n=5) and diabetic groups (n=5) analyzed in duplicate. *P<0.05 (Student’s *t*-test).

Cellular uptake of rhodamine 123 was analyzed and compared between groups (Figure 2A). VYS cells from the diabetic group showed a similar number of viable cells with active mitochondria (88.14±1.6) compared with the control group (94.1±1.3). However, an increased number of VYS cells with inactive mitochondria (nonviable cells) were present in the diabetic group (12.4±1.5 *vs* 5.3±1.1 in controls; P<0.05), indicating a reduction in Δψm. More activated caspase-3-positive VYS cells were present in the diabetic group (50.3±2.8) *vs* the controls (3.05±1.4; P<0.05; Figure 2B).

### DNA ploidy, cell cycle analysis and proliferation index

**Figure 3:**
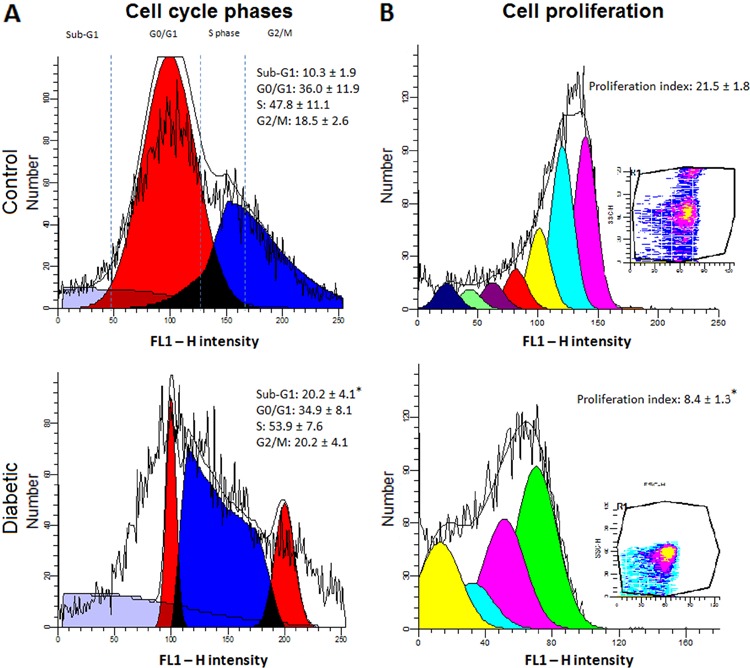
*A*, Cell cycle analysis showed the same variable distribution of visceral yolk sac (VYS) cells in all phases for both groups, except for the greater number of sub-haploid cells in the diabetic group. *B*, VYS cells showed a lower proliferation index in the diabetic group than in the control group. Results are reported as the means±SE of cells collected from five yolk sacs per animal of the control (n=5) and diabetic groups (n=5) analyzed in duplicate. *P<0.05 (Student’s *t*-test).

The number of diploid VYS cells was not different at gd 15 in the diabetic group (51.3±6.3) compared with the controls (62.9±5.9). However, fewer cells in the control group were aneuploid (38.4±5.7 *vs* 62.9±5.0, P<0.05). The results of cell cycle analysis showed the distribution of VYS cells in all phases to be similar for both groups, except for a greater number of sub-haploid cells in the diabetic group (20.2±4.1 *vs* 10.3±1.9, P<0.05) (Figure 3A). VYS cells showed a low proliferation index at gd 15 (8.43±1.3, P<0.01) in the diabetic group compared with the control group (21.5±1.8) (Figure 3B).

## Discussion

Maternal diabetes is associated with an increased incidence of congenital and placental malformations. Experimentally induced diabetes in pregnant animals, especially rodents, results in similar abnormalities to those observed in humans (2,14). In our study, the pregnant rats showed marked hyperglycemia and reduced mean fetal weight, which is a common finding in severe diabetes (15,16). Reduced fetal weight could be associated with yolk sac anomalies in rodents and humans (14,17).

The expression of cell markers CD90, STRO-1 and CD44 on VYS cells in both the control and diabetic groups confirmed the presence of MSCs in the rat VYS. CD90, also known as Thy1, is a glycosylphosphatidylinositol-linked protein involved in cell-cell and cell-matrix interactions, and some evidence indicates that CD90-expressing cells produce cytokines that modulate the hematopoietic stem cell microenvironment (niche) in bone marrow (18). The functional significance of increased CD90 expression by VYS cells is not fully understood but this could influence the mesenchymal niche and cell differentiation in diabetes as a result. Although, not mentioned by the Mesenchymal and Tissue Stem Cell Committee (19), STRO-1 is frequently used as *in vitro* marker of MSCs (20). Similarly, CD44 is expressed by MSCs and acts in cell adhesion, migration and homing (21,22).

Reduced OCT3/4 and CD34 expression was detected on VYS cells in the diabetic group while Nanog expression was not different between groups. In fact, OCT3/4 and Nanog are considered crucial for stem cell pluripotency and are usually expressed by MSCs (23). In contrast, CD34 is usually considered a negative marker for MSCs. However, there are some discrepancies between *in vivo* and *in vitro* studies concerning CD34 expression by many cell types, and it seems that the MSC negativity for CD34 is a cell culture-induced phenomenon and does not represent cell status *in vivo* (24). Moreover, CD34 expression is also present in hematopoietic progenitor cells. Therefore, the fresh isolation and culture of CD34 positive VYS cells is necessary to elucidate its role in VYS cell function and differentiation. Possibly, the hyperglycemic environment might reduce the pluripotency capacity of MSCs in the yolk sac to some extent, but further studies are necessary to evaluate this.

The immunopositivity for CD117, a marker for hematopoietic cell precursors, suggests that VYS cells at gd 15 retain the potential to generate hematopoietic lineages. Interestingly, increased CD117 expression was detected in the diabetic group. CD117 induces the proliferation and survival of primitive hematopoietic progenitors in the bone marrow, but its function in VYS hematopoiesis during diabetes is not well understood. Otherwise, the expression of VEGFR1 (Flt1) was not affected by the treatment. VEGF is essential for the survival of primitive erythrocytes (25), but the role of Flt1 during hematopoiesis is unclear because mice lacking the tyrosine-kinase domain of Flt1 have no obvious hematopoietic defects (26). In the yolk sac, early hematopoiesis is restricted to primitive erythroid, megakaryocyte and macrophage lineages (27) and, until gd 14, yolk sac macrophages have features of active phagocytosis (28). Besides the unaltered CD115 expression, the expression levels of CD14 and CCR2 were increased and decreased, respectively, suggesting that the hyperglycemic condition might influence cell differentiation in myeloid lineages.

An increased number of VYS cells with inactive mitochondria (unviable cells) was present in the diabetic group. An important study showed that mitochondrial function in VYS cells is maintained in normal pregnancy, even after the establishment of the chorioallantoic circulation (gd 12), becoming a supporting tissue until the placental functions are available completely (7). Therefore, the reduction of VYS mitochondrial activity in diabetes may be harmful for embryo nutrition. The loss of Δψm defines early stages of apoptosis preceding DNA fragmentation, production of reactive oxygen species (ROS) and increased mitochondrial membrane permeability (29). Indeed, hyperglycemia could induce intracellular damage by the generation of ROS (30) as superoxide has been implicated in the teratogenicity of culture media containing high glucose concentrations both *in vitro* and *in vivo* (31). Moreover, increased ROS could alter the mitochondrial transmembrane potential causing the permeability transition pore to open, leading to the leakage of proteins involved in apoptosis, such as Bcl-2, Bax and cytochrome C (32).

The increase in sub-haploid VYS cells in the diabetic group probably corresponded to apoptotic cells with fragmented DNA or condensed chromatin (33), as evidenced by the significant proportion of activated caspase-3-expressing cells in that group. Increases in caspase-3 and Bax expression levels were reported in whole embryos from days 9.5 to 11.5 after *in vivo* and *in vitro* exposure to a diabetes-like environment (32,34). Moreover, Reece et al. (35) showed increased Bax protein expression and decreased Akt kinase in yolk sac cells, suggesting that hyperglycemia triggers apoptotic signaling pathways and inhibits cell survival pathways. Yolk sac cells seem to be more sensitive to apoptosis under exposure to ethanol (36) and excess glucose (37), but the cellular mechanisms underlying apoptotic induction in those cells are still unclear and should be investigated.

VYS cells from diabetic animals showed low proliferation at gd 15. This was the opposite effect of maternal diabetes on placental development, which showed increased levels of proliferative markers (38,39), and the cell cycle inhibitor p57 (40) until gd 13 in the junctional and labyrinth zones of the rat placenta, indicating deregulated cell proliferation. However, no *in vivo* studies have been performed with rat yolk sacs at gd 15 for longer than 6 days after the induction of diabetes. In our model, alloxan was administered at gd 8 and the VYS samples were collected at gd 15; therefore, the reduction in cellular viability, increased level of activated caspase-3 cells and low cell proliferation confirmed the deleterious effects of diabetes on the physiology of the VYS and consequently on embryo/fetus development, even in late pregnancy.

In this report, we observed a negative effect of severe hyperglycemia on pregnancy and VYS viability, as evidenced by reduced fetal weights, and increased numbers of unviable and apoptotic VYS cells. In addition, the expression levels of VYS cell markers were altered in our model of diabetes. More detailed studies are required to explain the cellular and molecular mechanisms underlying yolk sac development and function during maternal diabetes.
